# Measuring and Modeling Behavioral Decision Dynamics in Collective Evacuation

**DOI:** 10.1371/journal.pone.0087380

**Published:** 2014-02-10

**Authors:** Jean M. Carlson, David L. Alderson, Sean P. Stromberg, Danielle S. Bassett, Emily M. Craparo, Francisco Guiterrez-Villarreal, Thomas Otani

**Affiliations:** 1 Department of Physics, University of California Santa Barbara, Santa Barbara, California, United States of America; 2 Naval Postgraduate School, Monterey, California, United States of America; 3 Sage Center for the Study of the Mind, University of California Santa Barbara, Santa Barbara, California, United States of America; National Research & Technology Council, Argentina

## Abstract

Identifying and quantifying factors influencing human decision making remains an outstanding challenge, impacting the performance and predictability of social and technological systems. In many cases, system failures are traced to human factors including congestion, overload, miscommunication, and delays. Here we report results of a behavioral network science experiment, targeting decision making in a natural disaster. In a controlled laboratory setting, our results quantify several key factors influencing individual evacuation decision making in a controlled laboratory setting. The experiment includes tensions between broadcast and peer-to-peer information, and contrasts the effects of temporal urgency associated with the imminence of the disaster and the effects of limited shelter capacity for evacuees. Based on empirical measurements of the cumulative rate of evacuations as a function of the instantaneous disaster likelihood, we develop a quantitative model for decision making that captures remarkably well the main features of observed collective behavior across many different scenarios. Moreover, this model captures the sensitivity of individual- and population-level decision behaviors to external pressures, and systematic deviations from the model provide meaningful estimates of variability in the collective response. Identification of robust methods for quantifying human decisions in the face of risk has implications for policy in disasters and other threat scenarios, specifically the development and testing of robust strategies for training and control of evacuations that account for human behavior and network topologies.

## Introduction

The development of new communication technologies enables rapid information dissemination and decision making among groups of individuals, but it also creates new challenges in the coordination of collective behavior. For example, the adoption of social networking technologies such as Twitter and Facebook can empower the masses but makes them hard to control [1]–[8]. More generally, the advent of contemporary network technologies has brought with it a new set of fragilities stemming from the complexity of human behavior: people rarely behave optimally, randomly, or uniformly, as often naively assumed in technological design and policy development.

Within the field of network science, the study of social networks plays an increasingly important role in method development and associated applications, with widespread implications in marketing, politics, education, epidemics, and disasters. Considerable effort is directed towards understanding how information diffuses through social groups [9]–[14], with particular emphasis on the role of news websites [15], blogs [16], Facebook [17], Twitter [18], and other social media [19], [20].

As information diffuses, individuals can display a range of decision making behaviors driven by new information. Phenomena of particular interest include (1) the dynamics of cascading behavior, which can explain how and why fads emerge [21] or rumors spread so quickly [22], [23], and (2) the role that individuals play as “spreaders” in facilitating the propagation of this behavior [24]–[26], or similarly the roll that “homophily” can play in abrogating uptake of a behavior [27]. Social epidemics, much like their biological counterparts [28]–[31], are often modeled as single- [32] or multi-stage [33] complex contagion processes [34]–[36].

Recent theoretical investigations have examined how this information exchange leads to collective action. In one class of models, individual agents occupy nodes on a network, and a set of rules defines information propagation dynamics and individual decision making behavior (e.g., see [23], [28], [37]). Complementary data driven investigations describe computational algorithms that begin to unravel rules for influence and decision making from large databases, such as Twitter, Facebook, and wireless communication networks (e.g., [6], [26], [38], [39]). In most cases the databases identify decisions that are made and delineate links between network members. However, information about the factors that drive human decisions, including individual observations, attention, history, personality, and risk perception is generally unavailable.

A topic of considerable interest is understanding how collective decisions may differ interestingly from individual decisions, with specific emphasis on the so-called “wisdom of crowds” (e.g., [1], [2], [30], [40], [41]). In this context, it remains to be shown at what scale group decision making might become more robust than that of individuals.

This paper focuses on a critical link between simulation studies and empirical observations of large scale networks. Specifically, we conducted a behavioral experiment involving a group of 50 individuals in a computer laboratory. Because human behavior is often far from what is predicted by idealized models, experimental observation in “live” and controlled environments are essential for improved understanding and modeling of social phenomena. Our work adapts the framework of Kearns *et al.* [42]–[45], who have conducted a series of “behavioral network science” (BNS) experiments that have focused on collective problem solving tasks, such as abstract graph coloring problems or economic investment games. These experiments, and similar experiments from other research groups, have demonstrated that “human subjects perform remarkably well at the collective level” in a number of tasks and scenarios, both competitive and cooperative [45]–[47]. However, disasters and other crisis situations often display the opposite effect [48]–[52]. Social interactions affect traffic flow [53], [54], and can lead to a “mob mentality” [55]–[57] that hinders evacuation and may lead to injury and violence. Moreover, associated spatiotemporal clustering of departure times can lead to traffic congestion and delays [58]–[60].

Therefore, in contrast to previous BNS research involving idealized, abstract games, our investigations involve decision making in a threat scenario. Specifically, our study is set in the context of an impending natural disaster, where each individual occupies a node in a social network and must decide whether or not to evacuate. The experiment is conducted for a sequence of time-evolving disaster scenarios. In each scenario, individuals receive real time updates from a centralized information source about the likelihood, severity, and timing of a disaster that threatens their virtual community. Individuals also receive social information regarding evacuation decisions of their neighbors, and availability of space in a virtual shelter. Thus, participants face a tradeoff in competing types of information (i.e., centralized broadcast information versus decentralized social information) in a laboratory setting that emphasizes risk and loss.

Compared to large data driven studies, the experiment provides a much more complete, quantitative set of measurements, enabling us to assess factors and isolate tensions that arise in human decision making. In addition to observing the ultimate evacuation decisions, our experimental setup allows us to monitor the behavior of individuals as they gather information. Prior to the experiment, we also assess individual personality profiles and risk attitudes using standardized tests. The ability to acquire this extensive set of static and dynamic measurements both prior to and during the decision making process allows us not only to look at how a population responds collectively to an evacuation threat, but also to try to understand whether individual variation in evacuation behavior within that population could be tied to risk preferences.

A primary outcome of this study is the identification of a decision model for evacuation behavior based on empirical observations. The model output fits the observations remarkably well and can be used to quantify individual differences in decision dynamics. The empirical model reduces the catalog of scenarios and observations to a few key parameters involving an overall multiplicative rate factor for evacuation, an average decision threshold based on the disaster likelihood, and variability about the average threshold, reflecting how consistently the decision making threshold was applied. The model enables us to isolate and compare two sources of urgency in the experiment that differentially impact observed behavior: time pressure for the evacuation decision and competition for shelter space. This empirical model stands in contrast to a set of models typically used in numerical simulations or large scale, data driven studies that treat decisions as random, optimal, or based on a threshold applied to a state variable representing opinion, which is updated by an assumed interaction rule (e.g., [21], [37], [53]–[56], [60], [61]).

While our experiment is admittedly well removed from a true natural disaster, it allows us to isolate and quantify tensions that arise in a crisis, in a manner that would not be possible during an actual event. Furthermore, the experimental design takes into account known psychological factors associated with risk perception, threat, and information processing [62]–[65]. A key component of behavioral network science is to use the observed human behavior as inspiration for the development of novel computational models of behavior, which can in turn be tested in future experiments. This spiral development of *model-experiment-model* or *experiment-model-experiment* may be used to develop optimal strategies for disseminating information during a disaster, and insuring sufficient allocation of resources for disaster response.

### Motivation

This work builds on three previous results involving collective decision dynamics in evacuation scenarios. The first is an assessment of evacuation routes and clearing times for a neighborhood threatened by wildfire [66], under the assumption of “best case” collective behavior as could be identified and implemented by a central authority. That is, individuals are assumed to evacuate exactly as directed and in a manner that maximizes the social welfare of the group as a whole. This idealized analysis captures the most salient features of evacuation behavior reported in a previous simulation-based study [58], and it provides an upper bound on collective performance, but it is not intended as a realistic prediction of real human evacuation.

The second result involves a detailed analysis of optimal “go” vs. “no go” decision making for an individual in the presence of a pending disaster [67]. Using a stochastic model that simulates the movement of a disaster (e.g., hurricane) through a bounded space toward a “target,” the decision to evacuate is modeled as a Markov decision problem. A dynamic programming algorithm is used to determine optimal decision policies which follow a multidimensional threshold form. The model is used to explore the tensions and tradeoffs in the decision to evacuate, specifically how optimal evacuation policies are affected by evacuation costs and disaster uncertainy.

The third result involves numerical simulation studies of collective decision dynamics where individuals, represented by nodes on a network, must decide whether or not to evacuate and are influenced by a one-to-many externally driven global broadcast as well as pairwise interactions on the social network [37]. In this context, an individual’s decisions are assumed to follow a threshold policy based on whether the individual believes that the disaster is sufficiently likely. By construction, it is possible to track both the diffusion of information regarding the likelihood of the pending disaster and the collective evacuation dynamics of the group. Our results indicate that social networks can help facilitate cohesive action among individuals, but that information transmission over the network can either facilitate or hinder action adoption. Moreover, we observe that cascading behavior is possible, especially if that information is binary, and that this depends in general on the influence of the global broadcast relative to the social network.

A primary motivation for the current experiment is to observe real human behavior in the context of a pending (albeit artificial) disaster, in the presence of both global broadcast information and social peer-to-peer information. The intent is to create a controlled setting in which all actions and observations are recorded prior to the decision, enabling development of a quantitative model that accounts for key drivers of decision making. These updated decision models can, in turn, be used in additional numerical experiments and analysis that ultimately informs the development of improved evacuation policies and strategies for real populations.

## Materials and Methods

On May 18, 2012 an experiment was conducted at the University of California, Santa Barbara (UCSB) in which 50 student participants within a virtual community each decided if and when to evacuate from impending natural disasters. All participants provided written informed consent, and the experimental protocol was approved by the Institutional Review Board of UCSB. The demographic composition of the participants was not released for publication.

Individuals participated in 47 scenarios (runs) that lasted one minute each. At the beginning of each scenario, each participant was given 100 monetary “points” that were at risk from a simulated disaster. During each scenario, participants were provided with information about the progression of the disaster, and they were offered the opportunity to evacuate from this disaster (a binding decision) and occupy one of a limited number of spaces in a virtual disaster shelter. Depending on their decision and the outcome of the disaster, they could lose some or all of their monetary points. The magnitude of the loss was a function of whether or not the individual successfully evacuated to the shelter, and whether or not the disaster struck. The total amount paid to a participant at the end of the experiment was a function of their cumulative score over the 47 runs. The running cumulative scores of all of the participants were ranked and displayed on a leader board at the front of the room. This allowed individuals to evaluate their strategy and provided a competitive incentive.

Prior to taking part in the study, the personality profile of each participant was measured using the Big Five Inventory (BFI-44) questionnaire [68]–[70], and the risk preferences of each participant were also measured in six domains (social, investment, gambling, health & safety, ethical, and recreational) using a Domain Specific Risk Attitude Scale [71], [72]. The Big Five Inventory is a commonly used set of 44 questions that enables the assessment of an individual’s personality along the following dimensions: extraversion, neuroticism, openness, conscientiousness, and agreeableness. The Big Five is used extensively in psychological research as well as in translational applications such as the assessment of learning styles and employee placement. The Domain Specific Risk Attitude Scale is used in psychological research to assess risk perception and risk behavior, to predict human behavior, and to develop policy in areas such as health and natural hazards. Administration of each questionnaire lasted approximately 7 minutes.

### Experiment Layout

The primary objective of this project was to understand the way in which individual decision makers use and share information, and how this information leads to collective action of the group as a whole. Of particular interest was obtaining insight into the influence of competing sources of information on individual and group behavior.

**Figure 1:**
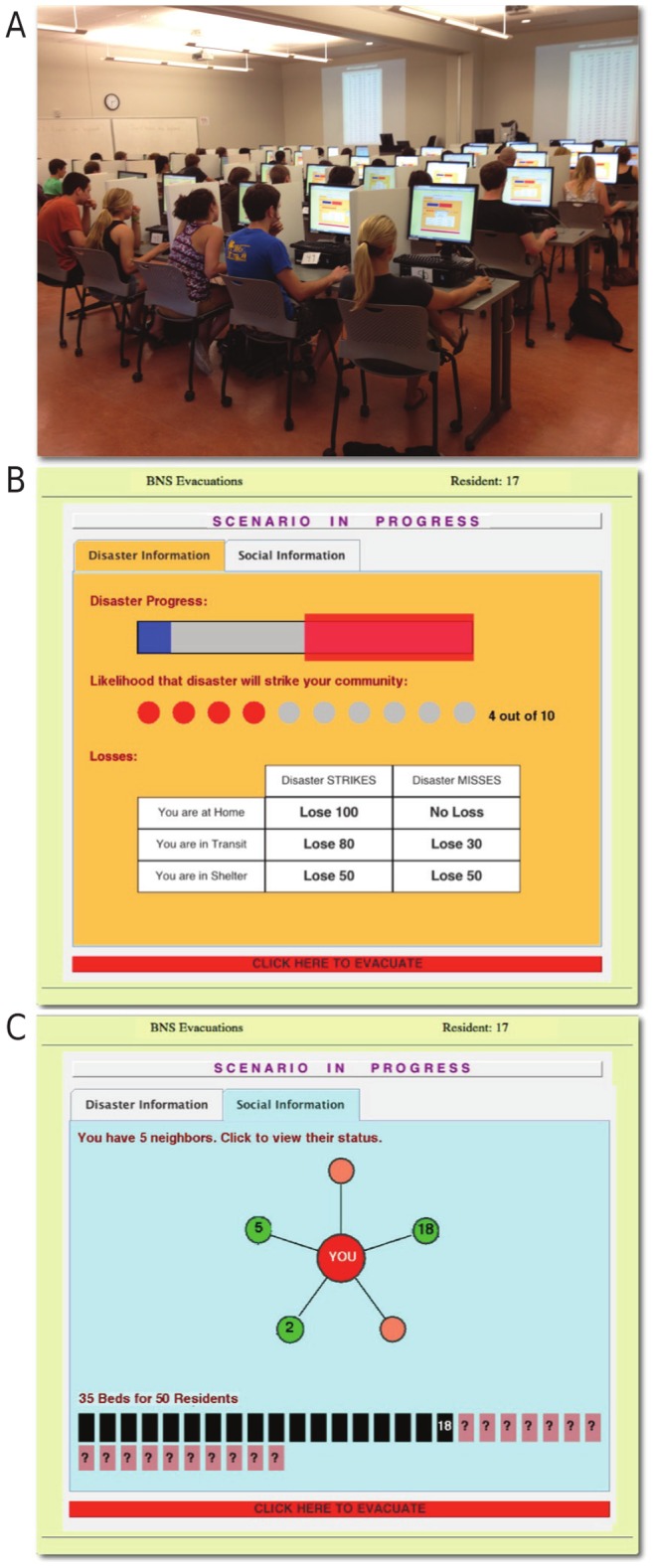
Overview of behavioral network science experiment. **A: Experimental setup at UCSB. B**: Disaster Tab, showing current status and loss table. **C**: Social Tab, showing status of neighbors; in this example, neighbors have claimed shelter spaces 2, 5, and 18, meaning that at least 18 of 35 shelter spaces have already been filled.

To reach these objectives, we employ an experimental setup derived from that of Kearns *et al.* [42]–[45]. We customize the computational framework and user interface to our evacuation problem. Each participant sits in front of a computer screen, see Figure 1**A**, containing two tabbed windows, labeled “Disaster Information” and “Social Information.” The participant may only view one window at a time and can switch between these two sources of information by clicking on the tabs.

The Disaster Information Tab (or simply, Disaster Tab), shown in Figure 1**B**, provides participants with information about the simulated time-evolving disaster. At the top of this tab is a disaster progress bar, which incrementally turns blue as time goes by; a red box around the scenario progress bar signifies the time window in which the disaster could strike. The likelihood that the evolving disaster will strike the community is presented in real time as the proportion of filled circles (e.g., 4 out of 10 filled circles indicates a current probability of 40%). A loss matrix shows how many points an individual will lose at the end of the current scenario depending on the outcome of the disaster and the individual’s final location. Finally, a button at the bottom of the Disaster Tab allows participants to evacuate. When an individual clicks the button, they transition from being “AtHome” to being “InTransit.” If there is still space available in the shelter, the individual immediately transitions to being “InShelter.” However, if the shelter is already full, the participant remains InTransit through the rest of the current scenario.

The Social Information Tab (or simply, Social Tab), shown in Figure 1**C**, allows the participant to query the status of neighbors in their social network by clicking on each neighbor’s node. If the neighbor is still AtHome, then the letter ‘H’ appears on the neighbor node. If the neighbor has evacuated, a subsequent click is required to identify this. If the neighbor is InTransit, then the letter ‘T’ appears. If the neighbor is in the shelter, then the shelter space (or “bed”) number that the neighbor occupies in the shelter appears. This value provides a lower bound on the number of beds occupied in the shelter and is also recorded in a shelter diagram toward the bottom of the Social Tab. The evacuation button located on the Disaster Tab is mirrored on the Social Tab to enable participants to make their evacuation decision irrespective of their current tab location.

### Psychometrics of Participants

#### Personality Metrics

**Figure 2:**
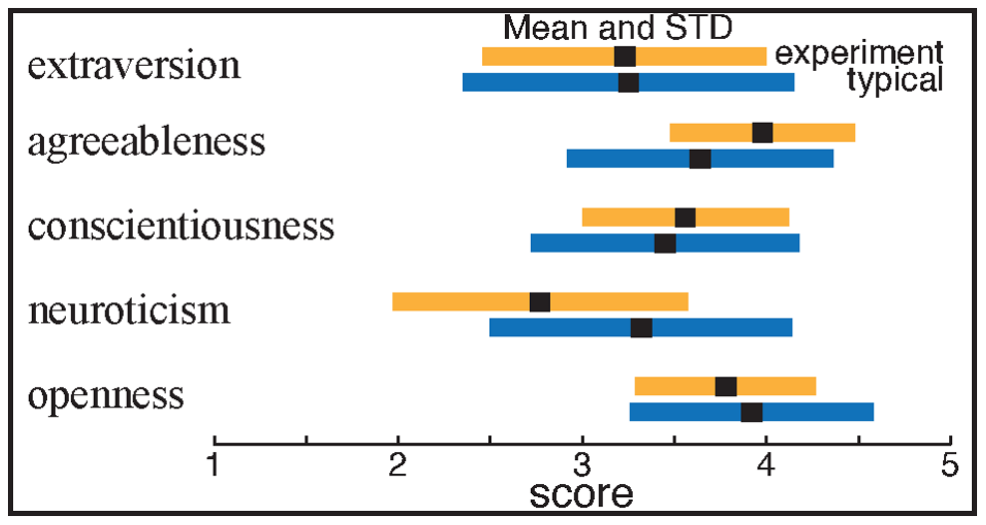
Mean and standard deviation (STD) for the Big Five Inventory scores calculated over all 50 participants (yellow). For comparison, we report the typical values estimated from 6076 individuals aged 21 (blue) [73]. The only significant deviation from typical scores was neuroticism, which had a significantly lower mean value.

The Big Five Inventory measures an individual’s personality based on five characteristics: extraversion, agreeableness, conscientiousness, neuroticism, and openness [68]–[70]. As shown in Fig. 2, the group of individuals that volunteered to take part in our experiment displayed similar personality profiles to the typical values for a similar age group [73], with the exception of neuroticism which was significantly lower than in the general population.

#### Risk Attitude

The risk attitude questionnaire scores both general risk attitude and specific risk types in the following domains: investment, health & safety, gambling, social, ethical, and recreational. The evacuation scenarios in this experiment were developed predicated on the assumption that individuals would be averse to the loss of monetary points (financial risk), and loss of life and property (health & safety risk). Participant responses to questions on the Domain Specific Risk Attitude Scale test ranged from “1” (Risk Averse) to “5” (Risk Seeking) with “3” indicating a risk neutral attitude. The general risk attitude distribution was risk averse (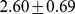). When segregated into the separate domains, the population displayed a range of risk attitudes summarized in Table 1.

**Table 1:** Risk Attitudes.

Domain	Mean	STD	Attitude Tendency
Social	3.49	0.57	Risk Seeking
Recreational	3.09	0.90	Risk Neutral
Gambling	1.59	0.77	Risk Averse
Health & Safety	2.65	0.64	Risk Averse
Ethical	2.02	0.56	Risk Averse
Investment	2.76	0.92	Risk Neutral

Risk attitude scores in 6 domains: mean and standard deviation (STD) calculated over all 50 participants.

### Scenario Simulation Mechanics

Our experimental setup had several key features designed to enable the isolation of external drivers and the identification of tradeoffs in decision mechanics. These features included a network structure linking participants and constraining information diffusion, time-evolving disaster trajectories, and scenario-to-scenario variation in shelter capacity, time pressure, and potential risk to monetary “points”. We describe these features in greater detail below.

#### Network Structure

In our experiment, a network structure enables participants to observe the actions of others. In each scenario, participants are assigned at random to a node in an underlying social network topology designed by the researchers. This allows an individual to have a different number of neighbors in each scenario, and for the number of neighbors to vary by individual in a single scenario. There were 8 networks used in the experiment: 3 “regular” ring lattice graphs, where each node was connected to nodes within a distance 1, 2, or 3, resulting in fixed node degree 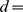 2, 4, or 6, respectively; and 5 “variable” graphs where nodes had degree 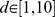 with an average 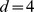. More specifically, the latter networks were generated as random graphs with specified degree sequence {1(×10), 2(×8), 3(×7), 4(×6), 5(×5), 6(×4), 7(×4), 8(×3), 9(×2), 10(×1)}, according to the algorithm specified in [74] and implemented in the NetworkX Python library [75]. Number of neighbors was varied to measure the affect on frequency of seeking social information. Different network structures were used as they predict different rates of information diffusion, with random networks having rapid diffusion, and regular lattice graphs having a slow rate of diffusion [76].

#### Disaster Trajectories

The disaster strike probability as a function of time 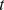, denoted by 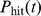, was generated in advance from a well-defined stochastic process previously studied in [67]; details of its construction can be found there. The process corresponds to a two-dimensional progression of a threat that moves toward a notional “target” with random lateral motion in one dimension and monotonic forward progression in the other dimension. The lateral motion is simulated with a range of step sizes limited by a prescribed volatility, while the forward motion may either have variation or step deterministically. We record a “Hit” (corresponding to a disaster strike) if the threat contacts a target, or a “Miss” if the forward motion causes the threat to pass the target without hitting. Participants can observe a truncated value of 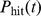 on the Disaster Tab which is updated every second, however the overall trajectory is not shown. There were a total of 23 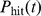 trajectories used in the experiment, with many of the trajectories repeated with different settings for other experimental variables.

#### Shelter Capacity

Scenarios varied in shelter capacity. There were 5 different shelter capacity scenarios: 50, 40, 30, 20, and 10 beds. When the number of beds in the scenario was less than 50 (the number of participants), individuals had to compete for access to these beds and could access information on the availability of shelter space through their social network.

#### Time Pressure

Scenarios varied in time pressure for an evacuation decision. When forward motion in the disaster trajectory model was deterministic, the disaster would either Hit or Miss at exactly 60 seconds. This type of time pressure is denoted “CertainTime”. For runs with variable time steps in the disaster trajectory model, the disaster could hit at any point between 30 and 60 seconds, with an end time that is not known in advance to the participants. We refer to this type of time pressure as “VariableTime”. The distinction between these types of scenarios could be observed by participants through the red box around the scenario progress bar on the Disaster Tab. These different scenarios were designed to test how temporal uncertainty affected evacuation strategies.

#### Potential Loss

Scenarios varied in potential risk to monetary “points”. At the start of a scenario, each participant is staked 100 points. The amount lost due to the disaster depends on the loss matrix, the outcome of the scenario, and by the individual’s location at the end of the run (AtHome, InShelter, or InTransit). Three loss matrices were used in the experiment and were based on underlying incentive structures designed by the researchers, with the values changing between runs acting to simulate varying disaster severity. The six entries in the loss matrix (seen on the Disaster Tab) correspond to the combination of the three end-state possibilities and the two disaster outcome possibilities. All loss matrices had a 0 point loss for an (AtHome, Miss) outcome, with increasing loss for (InTransit, Miss) and (InShelter, Miss). When the disaster hit, loss is minimized for the combination (InShelter, Hit), followed by (InTransit, Hit), and the most costly outcome is (AtHome, Hit). While one could envision many disaster scenarios where it would be more costly to be InTransit than AtHome, our modeling choice was motivated by InTransit resulting in distancing oneself from the disaster epicenter, and more generally, taking some action rather than none. Values in the loss matrix were deliberately chosen to prevent trivial solutions, such as always evacuate or always stay home, from being winning strategies.

#### Experimental Design

We used a nested experimental design to generate the permutations of model parameters–specifically network structure, disaster trajectory, shelter capacity, time pressure, and loss matrices–used in each run of the experiment. The resulting hierarchical structure guarantees that our experimental runs cover all potentially relevant parameter interactions.

**Figure 3:**
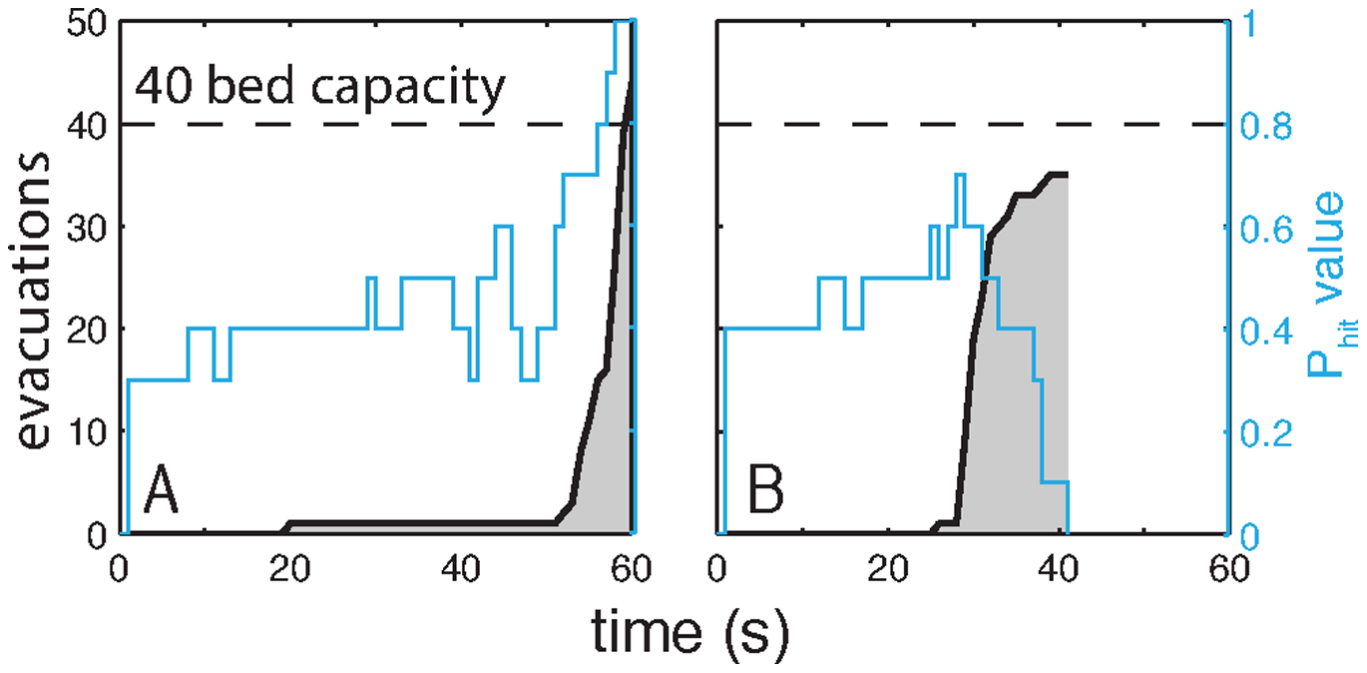
The collective evacuation behavior in two different scenarios. **A** (CertainTime): Participants wait until the end of the run to evacuate, waiting for more accurate information on the likelihood that the disaster will strike; some get stranded InTransit when the number of evacuees exceeds the shelter capacity. **B** (VariableTime): More than half the participants evacuate at approximately the 30 second mark, which is the first time that the scenario could end.

To summarize our setup and participant behavior, we plot the cumulative behavior for two evacuation scenarios in Figure 3. The overall behavior in each scenario can be observed by the interaction of the 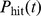 trajectory (in blue), the cumulative number of evacuations (grey fill), the number of available shelter spaces (dashed line), and the end time of the scenario. The scenario in Figure 3**A** is CertainTime while the scenario in Figure 3**B** is VariableTime. In both scenarios, there are 40 shelter spaces (beds) available for the 50 participants. In Figure 3**A**, we observe evidence of a stampede in which participants evacuated for limited shelter space toward the end of the scenario; some participants were left stranded in the state InTransit. In Figure 3**B**, we observe that a large number of participants evacuated at approximately the 30 second point in the scenario (the first time the run might end), but that the disaster did not happen.

## Results

The data collected during the experiment include every mouse click, for all 50 participants in each of the 47 disaster scenarios. From the data we can identify what each individual was seeing, when they were seeing it, and if and when they evacuated. This section describes empirical observations and statistical analysis based on these results, which is used to develop a quantitative decision model in the next section. Key variables include the strike probability (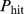) trajectory (Fig. 3 blue), the loss matrix, the number of beds in the shelter (Fig. 3 dashed-black), and time pressure for the evacuation decision.

### Participant Rankings and Scores

**Figure 4:**
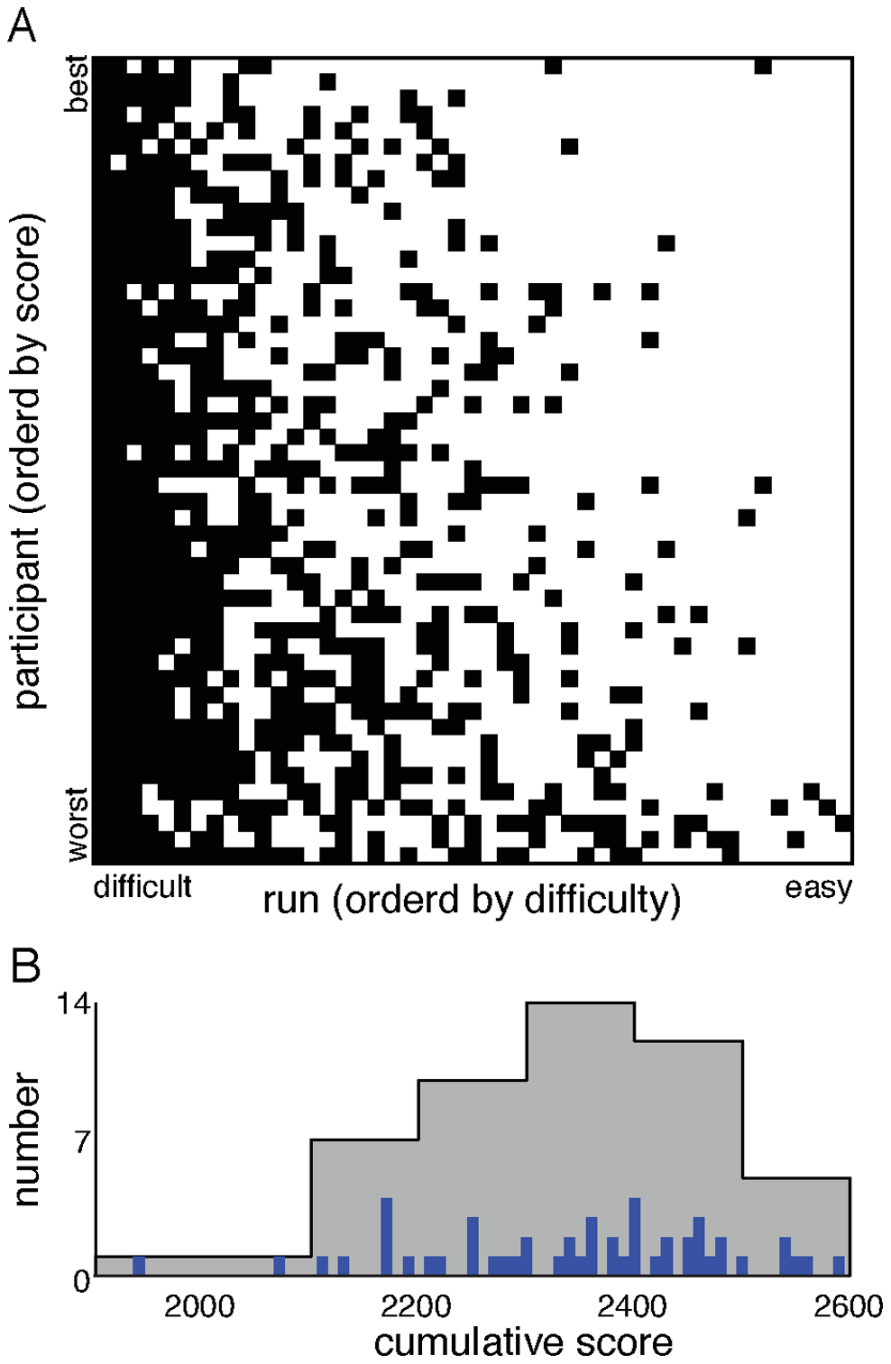
Success and distribution of cumulative scores. **A** shows successful decisions in white [(InShelter, Hit); (InTransit, Hit); (AtHome,Miss)] and unsuccessful decisions in black. The participants are ordered by cumulative score, with the highest scoring at the top. The runs are reordered with the most difficult run on the left. **B** presents a histogram of the cumulative scores (grey), with bars showing the exact scores in blue. The blue bars highlight the divergence of the most unsuccessful participant.

The success of each participant in each scenario is depicted in Figure 4**A**. We quantify a participant’s success using the total point score retained at the conclusion of the 47 runs. The three types of successful decisions [(InShelter, Hit); (InTransit, Hit); (AtHome, Miss)] are shown in white, while unsuccessful decisions are shown in black. In the “hardest” scenario (located towards the left-most side of the panel in Figure 4**A**), there were zero successes in the population, while in the “easiest” scenarios (located towards the right-most side of the panel) a single participant was unsuccessful in each run.

The distribution of cumulative scores is skewed: the lowest scoring participant is far below the rest (see Figure 4**B**). We analyze the differences in decision making patterns for different individuals in more detail in a later section entitled Individual Variation.

### Participants Focus on Disaster Tab

**Figure 5:**
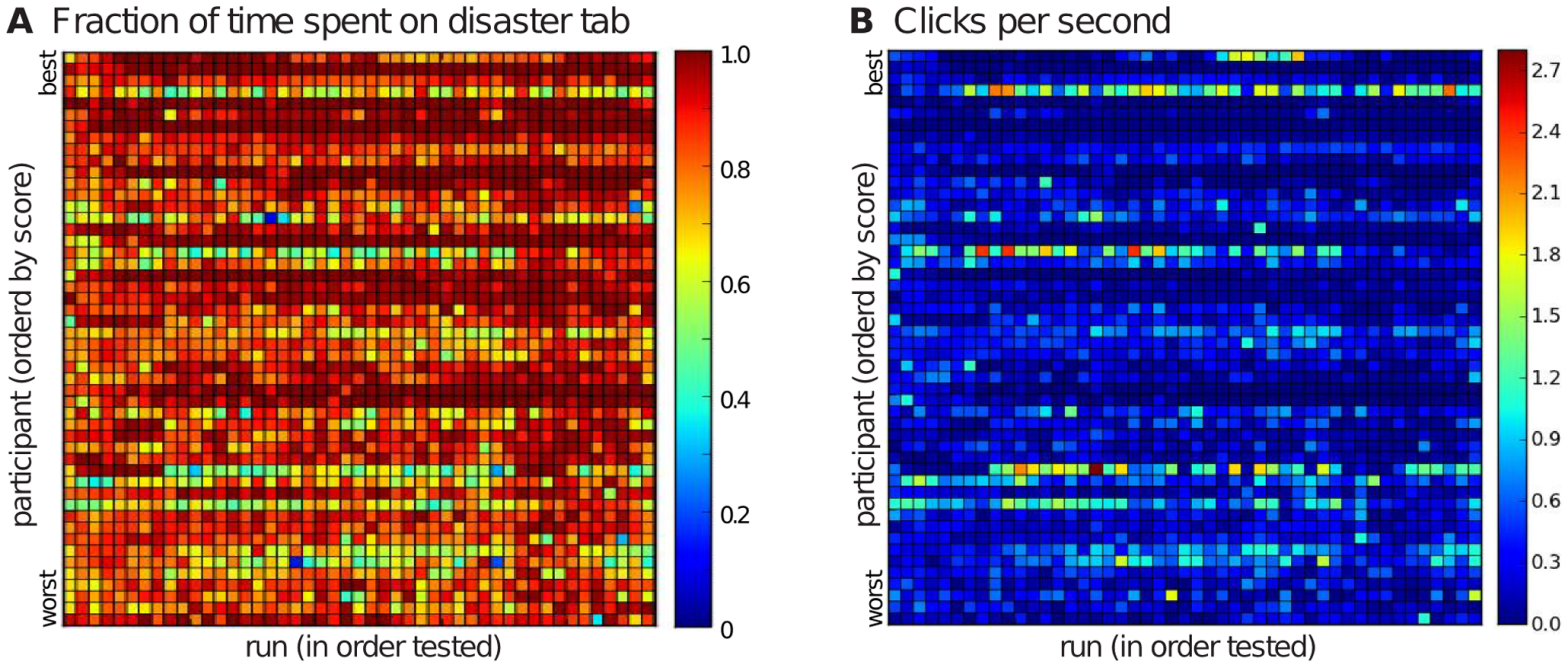
Participants spent the majority of their time on the Disaster Tab. (Frame **A**), but we can see those who spent more time on the Social Tab also had higher click frequency (Frame **B**) likely the result of trying to gain information on remaining shelter space.

Our results indicate that participants viewed the Disaster Tab more than the Social Tab. Individuals spent the vast majority of their overall scenario time on the Disaster Tab, and they made 99% of evacuation decisions while on this tab (see Fig. 5**A**). Although on average participants did not tend to spend as much time on the Social Tab, there was significant variation. We did not observe a significant relationship between time spent on each tab and performance.

### Clicking Behavior Links to SOCIAL Tab

Click frequencies for all participants in all scenarios are shown in Figure 5**B**, which lists participants by their overall performance (highest first). We can see from this figure that the higher click frequency individuals spent less time on the Disaster Tab and therefore more time on the Social Tab. The majority of participants displayed low values of clicking activity, indicating that they accessed social network information infrequently. We did not observe a significant relationship between click frequency and performance.

### Network Structure Drives Time Spent on Social Tab

**Figure 6:**
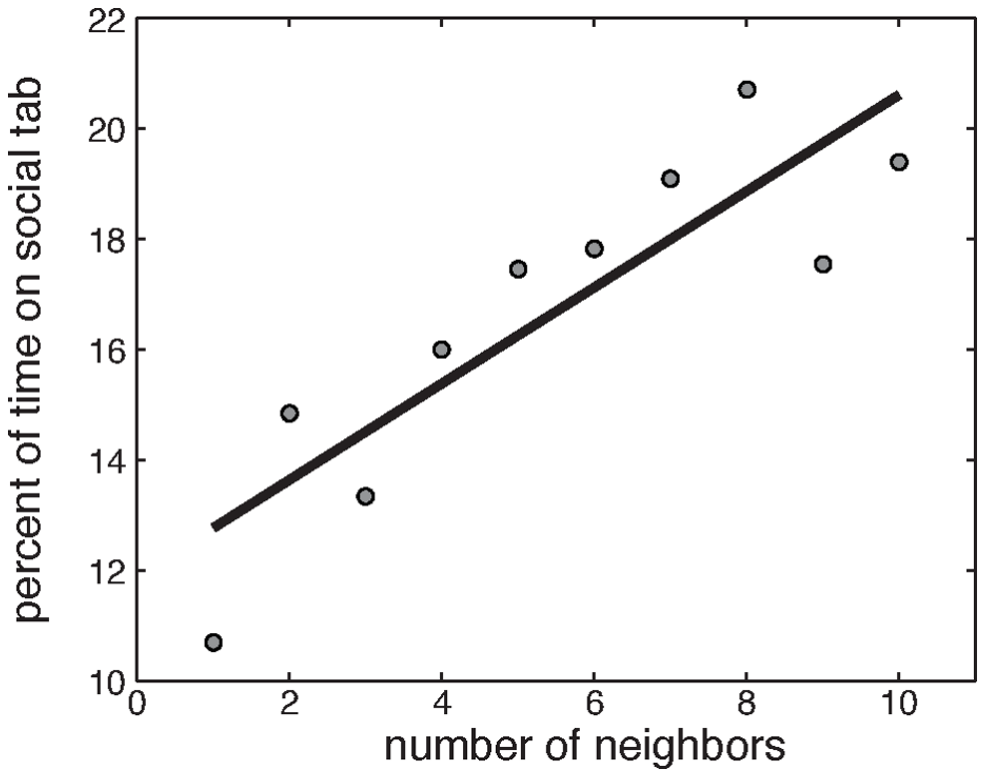
Relationship Between Number of Neighbors and Time Spent on Social Tab. The more network connections a participant had, the more time they spent on the social tab, with a Pearson correlation 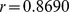, 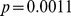.

The total number of neighbors a participant could have in any single scenario ranged between one and ten. Fig. 6 shows that participants with many neighbors tended to spend more time on the Social Tab than those with few neighbors. This result is intuitively consistent with the fact that highly connected individuals could gain more social information than less connected individuals, and might therefore be predisposed to spend more time on the Social Tab to obtain this information.

### Evacuation Decision Tied to Disaster Likelihood

**Figure 7:**
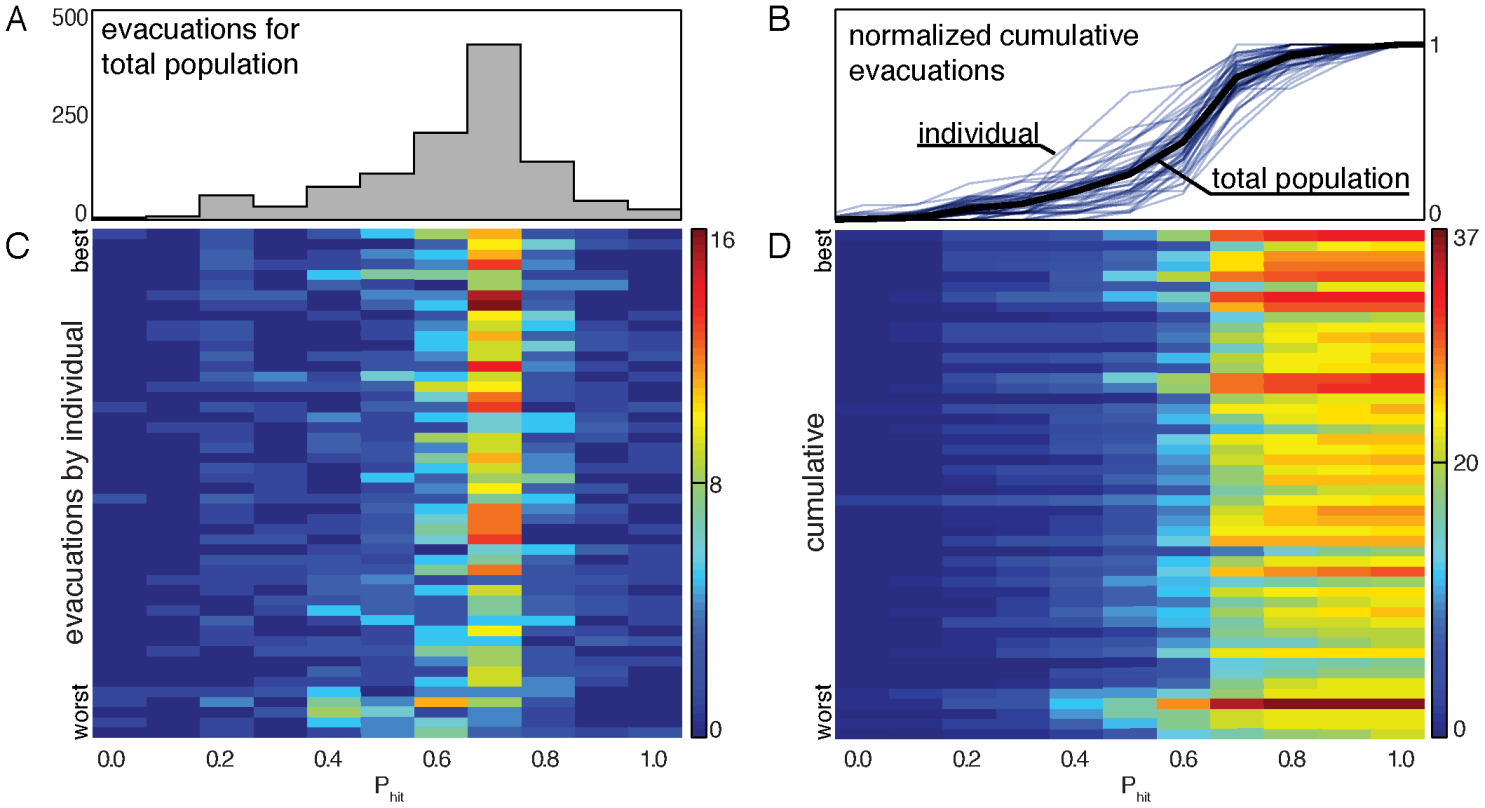
The distributions of evacuations as a function of 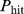. Frame **A** shows the numbers of evacuations at each of the eleven values of 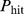. The distribution is peaked at 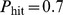. Frame **B** presents the normalized cumulative evacuation curves with individuals shown in blue and the population as a whole (the running sum of the distribution in **A**) in black. This provides a summary of the heterogeneity in evacuation decisions. Frame **C** shows the evacuations for each individual participant. Here we illustrate results for the highest scoring participant at the top and the lowest scoring participant at the bottom. We see a trend that the higher scoring participants evacuated more consistently at 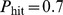, and the lowest scoring individuals have greater spread in the 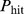 values at which they evacuated. Frame **D** gives the cumulative evacuations, a running sum of the data presented in **C**. We see that higher scoring individuals evacuate more readily, with the noted exception of the fourth worst scoring participant, who tended to evacuate much earlier than the others; a strategy that resulted in many unsuccessful evacuations.

Disaster likelihood values strongly influenced decision making, as shown in Fig. 7**A**. Here we see each observed evacuation grouped by 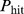 value at the time of evacuation. The distribution has a sharp peak at 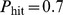. The cumulative distribution is shown in Figure 7**B** (black) and indicates that across all scenarios, about 90% of evacuations occurred before 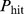 exceeded 80%.

### High Scoring Individuals Evacuate Frequently

We observed a significant correlation between score and number of evacuations at 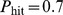 (Pearson correlation: 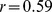, 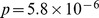). The lowest scoring individuals (see Fig. 7**C**, bottom) evacuate earlier and have a greater variation in the 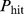 values at which they evacuate. In Fig. 7**D** we present the cumulative number of evacuations, a running sum of the the data in Fig. 7**C**. Here we observe a relationship between the total number of evacuations and score: highest scoring participants (top) are more likely to have a higher number of total evacuations than lower scoring participants (bottom). We confirmed this observation by calculating the Pearson correlation between score and total number of evacuations: 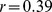 with 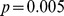. A notable exception to this trend is the fourth lowest scoring participant who also has the highest number of evacuations. Interestingly, this participant tended to evacuate much earlier than the other participants, resulting in many erroneous evacuations and therefore a lower cumulative score.

### Analysis

Following the experimental observations described above, our objective is to identify a model for evacuation decision making that can be used to quantitatively capture the main features of population level behavior (this section) and the heterogeneity of individual behavior (next section). The model will allow us to infer how the different experimental variables affect evacuation decision making. Our strategy uses data from the behavioral experiment to determine a decision model that depends on a few key state variables in the experiment (e.g., the probability of the disaster event 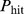). Based on summary statistics of evacuation behavior, we identify the functional form of the model and quantitatively estimate parameters. We then evaluate the accuracy of the model for predicting evacuations using state variables and detailed time trajectories from each individual run of the experiment. Our approach enables a concrete validation of our model, and provides direction for future experiments and large scale simulations of population behavior in similar scenarios.

Determining the dynamics of decision making strategies from the distribution of evacuations (Fig. 7**A**) is a complex problem that can be confounded by various factors including the distribution of 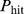 values observed by a participant and individual differences in reaction time. To account for these factors we introduce a rate model relating the number of participants evacuated to the number of participants AtHome, and determine how state variables such as 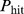 affect the rate.

As 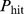 changes every second in our scenarios, it is natural for us to examine the data in one second intervals, within which 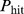 is constant. We then define two indicator functions that enable us to quantify the number of participants evacuated and the number of participants AtHome. First, we define the indicator variable 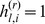 if participant 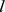 was AtHome at the start of the interval 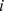 during run 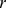, and 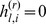 otherwise (i.e., the participant had already evacuated). Second, we define the indicator variable 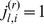 if participant 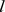 evacuated during interval 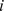 on run 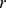, and 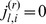 otherwise. These quantities are related by the equation:
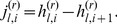
(1)


We approximate an individual’s decision to evacuate as a Bernoulli process in the following way. First we note that when 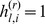, we can model the probability of evacuating during the interval 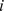 as a *rate*, denoted 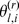, where 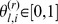. We treat the observed value for the indicator variable 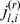 as one sample of an underlying stochastic process that can take a value of either 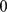 or 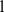. A single sample of the data provides a poor estimate of the rate 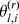. However, by modeling the data as a Bernoulli process, we can estimate the variance in rate, based on our limited number of observations. This approach enables us to derive a decision model without overestimating our confidence in small samples of data.

We hypothesize that 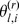 varies in a predictable manner according to a small set of state variables that capture the essential decision parameters in the experiment. To uncover these trends, we partition the data in a number of ways in this and the following section. In this section, we combine data for all the participants to obtain aggregate rates for the population as a whole, and in the following section, we consider heterogeneity in the evacuation rates of individual participants.

We begin by aggregating the data for specific disaster likelihoods 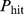, which in the experiment can take on values 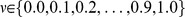. For each possible value 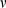, we determine the total number of intervals in the aggregate experiment where a participant who is AtHome observed 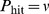:
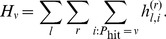
(2)


We likewise determine the total number of times such participants then evacuated:
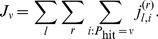
(3)


We use the uppercase 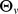 to indicate the evacuation rate for each value 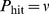. If we think of 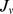 as a random variable (modeled as a sum of Bernoulli variables) given 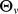 and 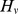, then 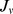 has a binomial distribution. Conversely, the likelihood of 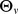 given 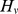 and 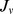, has a Beta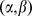 distribution [77], with parameters 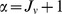 and 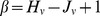. We thus *measure* rates from the data using the expected value of this Beta distribution:
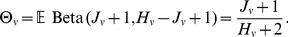
(4)


The standard deviation of these estimates is given by:
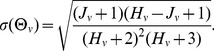
(5)


Given an abundance of data, the measured rate converges to the more intuitive fraction of evacuations 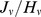. However, when data is limited the approach described above yields a more accurate description of the evacuation behavior.

**Figure 8:**
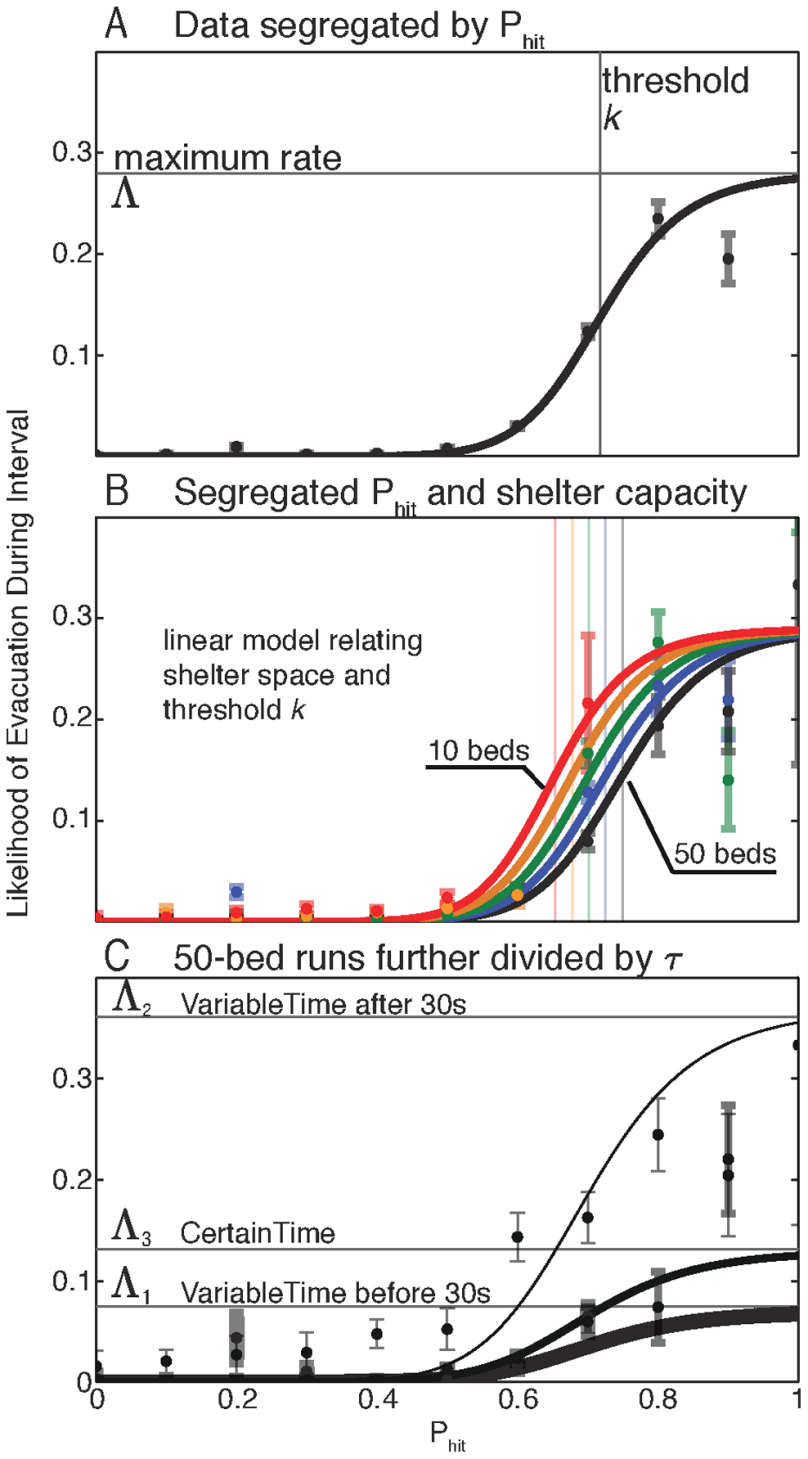
Model rate laws and their variation with shelter capacity and time pressure. In **A** we plot the measured rates for data partitioned only by 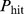 (black dots with grey bars for standard deviation), along with the best fit model (Eq. 6). In **B** we plot the measured rates for the data further partitioned by shelter capacity 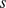, along with the best fit model where the mean threshold 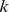 is a linear function of 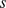 (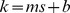). Line color indicates shelter capacity: 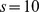 (red; top), 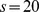 (orange), 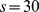 (green), 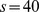 (blue), and 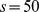 (black; bottom). Not all 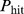 values were observed in all 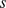 value scenarios. As bed number decreases, the rate curve shifts left, giving an increase in evacuation rate at the same 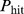. The model in **B** displayed systematic inaccuracies requiring partitioning the data into three different time scenarios (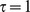 before 30 seconds in 30 second or greater runs, 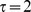 after 30 seconds in those runs, and 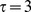 for 60 second runs). In **C** we plot only the 50-bed curves for the three scenarios and note that the rates for 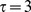 lie between 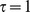 and 2.

Fig. 8**A** shows the estimated 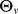 rates (black dots) associated with the 11 possible values 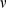 of the disaster likelihood 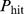. We observe that the rates increase approximately monotonically with 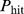 in a manner that is reminiscent of a Hill function [78]. We therefore model 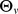 using the following functional form:
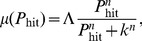
(6)which enables us to describe the decision making dynamics of the population using three parameters. First, 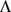 denotes the maximum evacuation rate; when 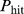 is large, 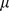 saturates to this value. 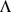 can therefore be used to estimate how quickly participants are able to react to rapidly changing conditions. Second, the threshold parameter 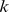 represents the half maximum value of 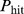, i.e., 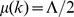. Third, the Hill-parameter 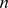 dictates the steepness of 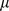 at 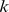. For large values of 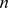 (e.g., 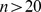), 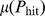 is threshold-like, being approximately 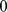 for 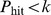, and approximately 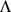 for 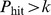. For smaller values of 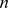 the transition is more gradual. Threshold policies have been extensively studied in previous work and are postulated to accurately characterize individual decision making behaviors in a variety of scenarios [21], [37], [79], [80].

All models used in the manuscript are fit to the data by evaluating the measured rates at each value 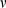 of the disaster likelihood to obtain 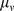. We then vary 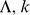, and 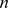 to maximize the expression:
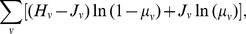
(7)a fit directly to the 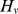 and 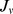 values, not the 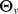 values. This expression is derived through maximum likelihood estimation [81] for Beta distributed measurements. The more common 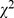 minimization for curve fitting is similarly derived from maximum likelihood estimation for Gaussian distributed measurements [81], and our formula serves the corresponding role.

Fitting our model to the measured rates in Fig. 8**A**, we obtain 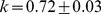, 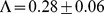, and 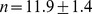. The standard deviations reported here were obtained via bootstrapping [81] where we constructed synthetic data sets by randomly selecting 47 runs with replacement from the original data, then aggregating the data and fitting the model to the synthetic data using the method described above. The best fit model is plotted in Fig. 8**A** (solid black line). For most values of 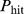, we find that this model accurately captures the observed behavior. However, we also observe systematic variations between the model and the experimental data. One set of variations appears to stem from shelter capacity while the other appears to stem from temporal urgency for the evacuation decision.

To examine the role of shelter capacity 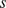 in decision making, we aggregate the data for each of the 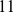 disaster likelihoods 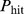 at each of the 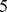 values of shelter capacity 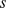. We adapt our use of the subscript 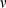 to now indicate this finer-grained aggregation into 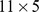 sets of data. The measured rates confirm our expectation that evacuation rates were high when shelter space was scarce and low when shelter space was abundant (see Fig. 8**B**).

To model the role of shelter capacity in modulating the average form of the evacuation decision, we consider two families of Hill functions based on our previous fits: one family drawn from variations in 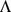 and a second family drawn from variations in 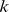. To guide our choice between these two alternatives, we consider optimal decision making behavior. If shelter space is abundant and information is precise, the optimal evacuation decision rule will be a threshold-like function in which the value of the threshold is just below 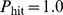. This behavior ensures that the individual evacuates when there is near certainty that the disaster will hit the community. If instead there is very limited shelter space and the costs of the two possible incorrect decisions are equal, the expected evacuation decision rule will also be a threshold-like function, but in this case the value of the threshold will be just above 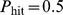. This behavior ensures the best chance of getting a bed in the shelter, which is the lowest loss associated with a wrong decision.

Because the threshold value appears critical for optimal decision making behavior in scenarios of both abundant and scarce shelter space, we choose the family of Hill functions obtained from varying 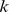. We find that the following linear model of 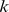 versus 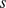:
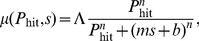
(8)fits the data well. In Fig. 8**B**, we show the set of curves extracted for the best fit to the model in (8) alongside the raw empirical data. The best fit values for 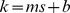 are 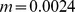 and 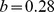.

To test the accuracy of this model and to identify systematic differences between the best fit model and the data, we compared the predictions of this model to the data, and found a systematic trend whereby we overestimate the number of evacuations occurring prior to 30 seconds in VariableTime runs and underestimated the number of evacuations occurring after 30 seconds in those runs. The difference between actual and predicted evacuations was profound and the shift between overestimating to underestimating was abrupt, shifting at exactly the 30 second mark in nearly every VariableTime run. These results show that an individual’s behavior is additionally influenced by temporal urgency.

To quantify the effect of temporal urgency, we extend our model in the following way. As in the previous versions of the model, we aggregate the data for each of the 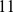 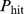 values at each of the 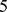 values of shelter capacity 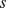. However, in this case we additionally aggregate data for the following 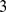 separate cases with differing temporal urgency: prior to 30 seconds in VariableTime runs (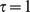), after 30 seconds in those runs (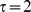), and all data in CertainTime runs (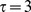). We again adapt our use of the subscript 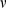 to now indicate this even finer-grained aggregation into 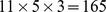 sets of data.

To determine if temporal urgency had a more significant effect on 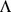 or on the threshold parameters (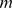, and 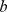), we fit the model equation in Eq. 8 independently to the 3 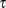 cases. From these fits and the confidence intervals on the parameter estimates we were able to determine that the variation of 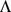 with temporal urgency was more significant than the variation of 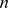, 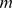, or 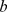. We therefore constrained variation with temporal urgency to 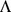, adopting a six parameter model:
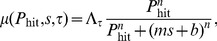
(9)which has three 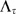 valuse. The best fit values are presented in Table 2.

**Table 2:** Parameter Estimates.

Parameter	Symbol	Value	STD
Hill-coefficient	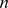	9.3	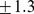
Maximum rates:			
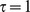	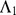	0.07	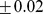
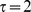	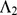	0.37	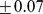
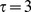	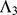	0.13	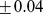
Threshold parameters:	( 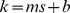 )		
Offset	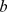	0.60	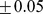
Proportionality const.	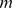	2 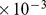	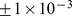

Parameter Estimates for the the model in Eq. 9, with standard deviations obtained via bootstrapping [82].

Figure 8**C** illustrates the measured rates and model curves for a characteristic subset of the data (runs with 50 beds) for each of the three time windows (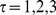). For this partitioning of the data both the first 30 seconds of VariableTime runs (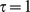) and the full 60 seconds of CertainTime runs (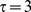) are described by similar low evacuation rates 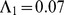 evacuations/second and 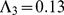 evacuations/second, respectively. Both of these are significantly smaller than the corresponding rate 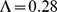 evacuations/second for original aggregated data (Figure 8**A**) as well as the rate 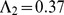 evacuations/second observed after 30 seconds in the VariableTime runs (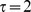). The increase in rate during the uncertain window in the VariableTime runs reflects a high temporal urgency associated with a disaster that could strike at any moment. It also suggests participants will respond quickly to changing 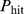 values under these conditions.

The relatively low values of 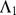 and 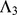 are likely due to the fact that in these cases the disaster strike is only possible in the last time increment of these partitions, a low temporal urgency. In each case, urgency increases towards the end of the interval, and this occurs to a greater degree for 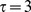 (CertainTime) than for 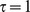 (first time window in VariableTime). In CertainTime runs, the scenerio terminates at exactly 60 seconds, so in this case the last observed 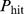 value describes the likelihood of a strike *at* 60 seconds, whereas in the first 30 seconds of the VariableTime runs the value of 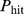 at the end of the interval reflects the probability of a Hit not necessarily in the next time increment, but rather at some time within the uncertain 30 second window. We expect this distinction underlies our observation that 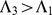.

### Simulations

We test our decision model by using it to simulate evacuation behavior for the 47 scenarios in the behavioral experiment. The appropriateness of our model can then be quantified by the difference between simulated and observed behavior, with small differences indicating that our model could be used as a generative model in future numerical studies.

In the experiment, each scenario is characterized by a shelter capacity 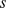 and time pressure 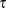, as well as a prescribed sequence of disaster likelihood values 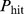. Using our decision rule, we can compute the expected rate of evacuations at each instantaneous value of 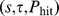. If we initialize every simulation with 50 individuals at home (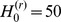), we can compute the expected number of people AtHome in each interval 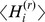 using:
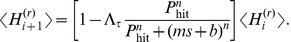
(10)


**Figure 9:**
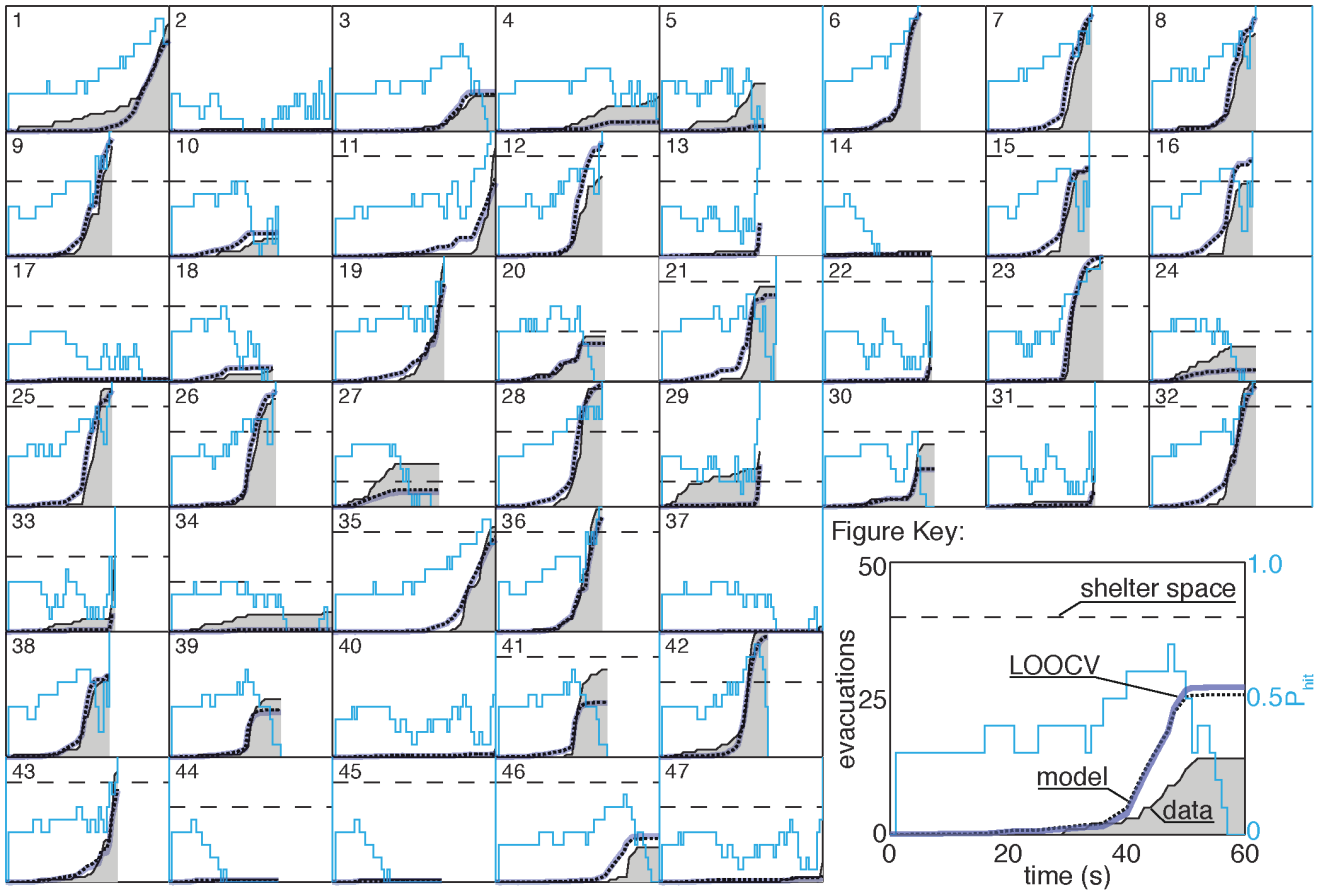
A comparison between data and simulation for the 47 scenarios and the best fit six-parameter model defined in Eq. 9. At each second the 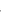 value (blue), the shelter capacity, and the time scenario determine the rate used in the simulation, and the expected number of evacuations is calculated. The model was fit to estimated rates (Eq. 4), not to the time series data shown here. This extends the ability of the model to predict untested scenarios. To illustrate the predictive capability we also plot the *leave-one-out cross-validation* (LOOCV) predictions (violet curves). If the model were over-fit, the LOOCV curves would have significant deviation from the full model. The reduction from 2820 rates in the data to a six-parameter model generated a model with surprising accuracy. The following runs had identical 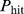 trajectories: (1,35), (3,46), (8,25), (9,36), (12,26), (13,29), (14,44,45), (15,16,38), (19,43), (22,31,33), (34,37), (39,41), (40,47).

In the paragraphs below, we comment briefly on several key results from our simulations (see Fig. 9).

#### Decision model accurately describes experimental observations

In the majority of scenarios the simulated behavior has very little deviation from the observed behavior. This result is striking because our model aggregates the data over all participants over all scenarios to a reduced set of six parameters, with no time resolution aside from separation into the three bins associated with the different time pressure variables. In the majority of scenarios the simulated evacuation behavior is qualitatively, and in many cases quantitatively, matched to the observed behavior of experiment participants.

As a check that we have not over-fit the model, we have performed a *leave-one-out cross-validation* (LOOCV) [83], where for each of the 47 runs, we exclude the data from that run, and see how the model trained on the other 46 runs predicts the outcome. The LOOCV results (Fig. 9, violet curves) were nearly identical to the predictions of the full model (Fig. 9, dotted curves), indicating that the model is not over-fit. This result also suggests that the model will predict the outcome of other scenarios with the same accuracy of the simulations shown here, assuming that the 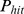 trajectories are created using the same rules.

We begin our description of Fig. 9 with the three runs where participants had the most success, 36, 44, and 45. As can be seen here and in Fig. 4**A** (far right), all but a single individual made the correct evacuation decision in these runs. In run 36, the disaster had a very predictable trajectory, gradually increasing in 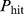 before eventually striking. In runs 44 and 45, the disaster had a poor likelihood of striking and 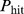 decayed fairly rapidly. In contrast, the most difficult run was number 42. The 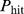 trajectory in this run peaked at 0.9 before the chance of a disaster strike rapidly decayed and the run ended with a Miss. As can be seen here and in Fig. 4**A** (far left) every participant was left either InShelter or InTransit.

#### We observed sub-optimal decision making

In general, the optimal decision to evacuate in a given scenario depends not only on the likelihood and volatility of the underlying disaster process, as well as on the loss matrix, but also on the shelter capacity and the decisions of other individuals. However, scenarios 1, 2, 3, 4, 37, and 40 are unusually simple in that participants knew that these scenarios would each last exactly 60 seconds, and that there was adequate shelter capacity for all participants. These two simplifying factors ensured that the actions of other participants had no direct effect (though they could presumably influence behavior, e.g. peer pressure). In these scenarios, it would be optimal to wait until immediately before the potential disaster strike to evacuate. As Fig. 9 indicates, in scenarios 1, 3, and 4, participants did not follow the optimal strategy; rather a significant number of participants evacuated well before the end of the scenario. In fact, many participants evacuated after only approximately 30 seconds. This behavior proved costly for them in scenarios 3 and 4. Scenarios 2, 37, and 40 are less conclusive because the strike likelihood 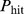 in these scenarios never exceeded 0.5 (and the disaster did not hit), making it relatively easy to decide not to evacuate.

#### Participant behavior adapts over time

By construction, several scenarios contained identical 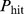 trajectories but differed in other parameters. Among these “repeated” disasters, we observe evidence of learning with regard to time pressure. In runs 1, 3, and 8 there were some unnecessarily early evacuations, but participants waited longer to evacuate in the corresponding runs occurring later in the experiment (runs 35, 46 and 25).

This observed adaptation could be explained either by effects of time pressure or by effects of strike likelihood. To determine the dominant driver of the adaptation, we compared the evacuation rates in runs 1–8 with those in runs 37–40 to determine whether there was evidence for adaptation in decision making strategies. While these runs differed in strike likelihood, the measured rates observed in the two groups did not show a significant change at high 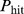 values. This suggests that although participants seemed to adapt their strategies in relation to time pressure, they did not adjust their behavior in relation to strike likelihood.

#### Amplified sensitivity to lowest shelter capacities

In each of scenario runs 27 and 29, shelter beds were scarce (10 beds for 50 people) and more participants evacuated early in the scenario than our model predicted. It is possible that either (1) our linear model of the variation of the threshold 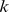 with shelter capacity 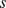 is inadequate when shelter space is very scarce, (2) that time pressure affects player behavior before 30 seconds in VariableTime runs with low shelter capacity, or (3) the participants were reacting to each of these scenarios also immediately following runs in which a large number of individuals evacuated after the shelter was full, leaving those individuals stuck InTransit (runs 26 and 28). The early evacuations in runs 27 and 29 could therefore be a reaction to participants being caught InTransit in the previous run. We are unable to discriminate between these three possibilities with this data set; we leave this for future work.

### Individual Variation

Our success in identifying a decision making model that captures the observed collective evacuation behavior in the experiment led us to test whether a similar method could differentiate between individual decision making strategies. In the previous analyses, we combined data for all of the participants, which enabled us to fit the model to several experimental variables. Because the evacuation data for individual participants is relatively sparse, here we focus exclusively on the influence of the disaster likelihood 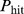 in decision making and do not separately consider the effect of shelter capacity or time pressure.

**Figure 10:**
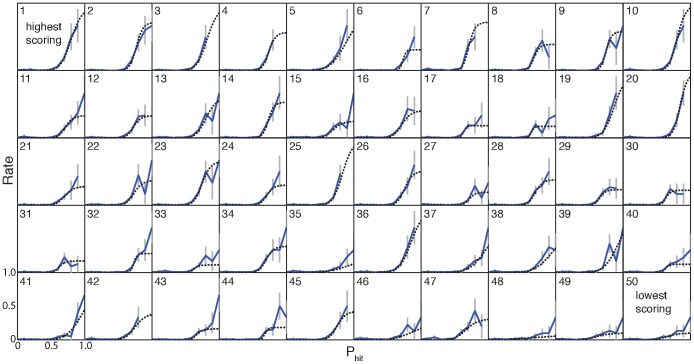
A comparison between the decision making model and data from the behavioral experiment for each participant, ranked according to cumulative score. Evacuation rates for each individual at each 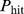 value were measured using Eq. 4. These values are plotted in blue accompanied by the estimated standard deviations for each point (grey bars) calculated based on Eq. 5. Hill functions were fit for each individual using the routine described in Eq. 7 (dotted black). Higher evacuation rates tend to result in higher scores. The fits give a significant correlation between evacuation rate 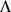 and score (Pearson 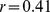, 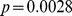). Moreover, individuals who evidencing higher financial risk attitude scores (i.e., more risk seeking) have higher thresholds for evacuation 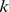 than individuals evidencing lower financial risk attitude scores (Pearson 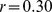, 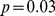).

To extend the collective decision making model to individuals we estimated the evacuation rates for each participant at each 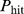 value using Eq. 4. We show this data in Fig. 10, where individuals are ranked by score from highest scoring (top left) to lowest (bottom right). Some individuals had as few as 9 measured rates, as they consistently evacuated before 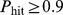 (see truncated curves in Fig. 10).

Comparing the raw data in Fig. 10 for individuals with the corresponding measured rates for the aggregate population shown in Fig. 8 illustrates an interesting deviation in the measurements at high values of 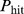. For the aggregate population there is a significant and somewhat counterintuitive drop in measured rate from 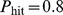 to 0.9; the value of the measured rate represented by the data points at 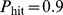 lies below the value represented at 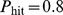. However, while non-monotonicity is observed on the scale of individuals the trend is not systematic (see Fig. 10). The difference between the population and individual fits suggests that the observed drop in the measured rate at high 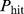 in aggregate data is driven by heterogeneity in the population. Participants with high evacuation rates tend to leave before 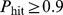. Those who remain and observe high values of 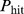 typically display low evacuation rates, thereby biasing the summary rates measured at the population scale.

To capture individual decision making strategies, we fit a three-parameter Hill function (Eq. 6) to each individual’s measured rates using Eq. 7. As shown in Fig. 10, the best fit models based on the Hill function capture the measured rate curves of each participant with striking accuracy.

#### Higher evacuation rates accompany better performance

The wide range of participant decision making behavior is clearly visible in Fig. 10. The variability is especially apparent when we compare the highest scoring individuals with the lowest scoring individuals. The highest scoring participants exhibit rates that increase sharply and monotonically, approximately beginning at 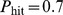. The lowest scoring individuals rarely evacuate; we observe flat evacuation rate curves, with measured rates that are relatively much lower and less systematic in their variations compared to high scoring individuals. As is apparent from the accuracy of the fits, this distinction is well captured by our model.

**Figure 11:**
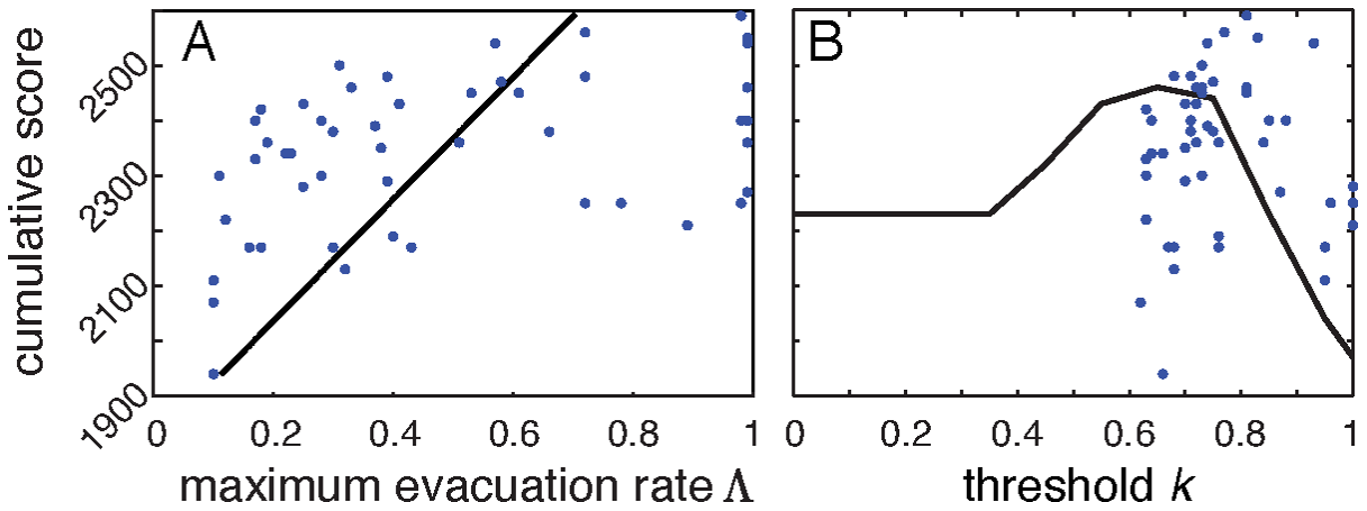
Best fit models provided values for the maximum evacuation rate 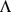 and threshold parameter 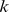 for each individual. A The distribution of 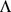 values across participants spanned almost the full range from 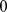 to 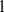. Here we observe a significant correlation between 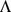 values and cumulative score across participants (Pearson 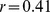, 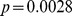). This result provides statistical support for the apparent tendency for high scoring individuals to also display higher rate values (see Fig. 10). B cumulative score vs threshold parameter (blue dots) had no significant linear correlation. A strict thresholding strategy (black curve), where a model player would immediately evacuate once 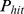 exceeded their threshold, helps to explain the lack of linear correlation. If a threshold is set too high, it results in many AtHome Hits while too low results in InShelter Misses. There is a maximum cumulative payment for strict thresholding between 0.6 and 0.7. We see that participants typically had thresholds above this range and scored higher than the expected payoff (blue dots). This is likely a result of participants incorporating time pressure and scarcity into their decisions, having reductions in score from a low 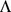, and variability in having a non-threshold (low 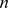) strategy.

A fundamental goal of our experiment was to identify psychological and behavioral predictors of individual performance. First, we ask whether parameter values from the best fit models on individual participants could be related to behavioral performance in the experiment. The best fit models yielded rates 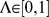, with values for every individual displayed in Fig. 11 **A**. Overall, we observe a significant positive correlation between the maximum evacuation rate 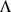 in the best fit models and cumulative score (Pearson 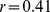, 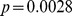; see Fig. 11**A**). We speculate that the maximum evacuation rate could be related to a participant’s fundamental reaction time. If true, our results suggest that participants who can react quickly to rapidly changing conditions in their environment are more successful in the experiment.

As expected, we do not see a significant linear correlation between cumulative score and threshold parameter 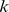. This results from a mid-range value of 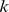 having an optimal effect, with low thresholds resulting in erroneous evacuations, and high thresholds resulting in disaster strikes while AtHome. To illustrate this optimum we plot the cumulative score varying 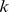 for a strict threshold model (i.e. high 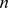, 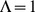) in Fig. 11 **B** (black curve). Here we see that the maximum cumulative score for this type of decision model is at 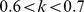. This calculation does not take into account shelter space or time pressure, which individuals (blue dots) used in order to get improved scores. The population as a whole had a higher threshold parameter (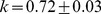) reflecting the use of this additional information in obtaining higher cumulative scores. Decisions also had a considerable stochastic component for low 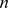 and 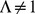, giving more variability in scores.

#### Similar decision models can produce different scores

It is noteworthy that some low and intermediate scoring participants display reduced (binned) decision statistics, and consequently decision model parameters, that are almost identical to those of the highest scoring participants. For example, participants 1 and 36 have very similar decision models but very different scores (2590 and 2270). This result indicates that in some cases similar decision making strategies can produce very different performance outcomes.

Our decision model reduces the data to a single scenario parameter (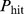) and therefore fails to capture the other features that are likely to be important in distinguishing between individuals such as timing of the decision. Our data on the population scale suggested that time pressure and shelter capacity are important variables and likely have similar importance on the scale of individuals. By comparing the detailed time evolution of individual runs, we observe instances where higher scoring participants tended to wait longer before evacuating than lower scoring participants, a more successful strategy.

While we are unable to quantify with significance these effects in the current experiment due to limited data, our model provides a tool for estimating the quantity of data needed to robustly quantify these parameters in driving individual decision dynamics.

#### Individual variation in performance may be tied to risk preference

We hypothesized that risk attitude could be a significant factor in the evacuation decision making of an individual and therefore affect the overall performance of participants. For the participants in this experiment, we found that cumulative score was significantly correlated with health & safety risk attitude (Pearson correlation: 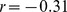, 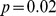) but not with financial risk attitude (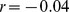, 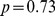). These results indicate that individuals that were more averse to health & safety risks (and therefore potentially more susceptible to the specific influences associated with an evacuation decision scenario) performed better than those that were less averse.

We then tested whether risk scores in either the health & safety domain or the financial domain were related to individual differences in decision making strategies. We estimated an individual’s general financial risk attitude by averaging their scores from both gambling and investment risk domains [71], [72], and we estimated their overall performance using the cumulative score. We found a significant relationship between 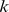 and risk score in the investment domain (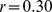, 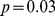), indicating that individuals with higher decision thresholds tend to have more risk seeking attitudes. We interpret this result with caution due to the possibility of Type II errors in the large number of tests performed (3 risk scores and 3 best fit model parameters = 9 tested correlations). However, a correlation between these two variables is plausible; it suggests that participants who tolerate more financial risk are more likely to wait until the disaster is imminent before evacuating.

An interesting question is whether the observed correlation between risk attitude and performance was consistently observed over the population or whether it was driven by a subset of individuals. From a psychological perspective, one meaningful segregation of individuals into groups is a partition based on the consistency of individual risk preferences across domains. Individuals with consistent risk preferences across domains often display different personality traits – which could directly lead to differences in behavior – than those with inconsistent risk preferences across domains [84]. To estimate the consistency of risk attitudes we computed the standard deviation 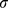 of mean scores across the 6 risk domains. We separated participants into a “consistent” group, composed of those individuals with 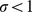 (N = 31), and an “inconsistent” group, composed of those individuals with 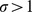 (N = 19). The observed correlation between performance and health & safety risk attitude appears to be driven by individuals with inconsistent risk attitudes (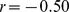, 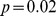) rather than by individual with consistent risk attitudes (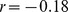, 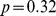). This suggests that individuals with domain specific risk attitudes might tune their behavior more closely to the risk structure of the experiment.

## Discussion

The behavioral network science experiment reported in this paper quantifies several key factors influencing individual evacuation decision making in a controlled laboratory setting. The experiment includes tensions between broadcast and peer-to-peer information, and contrasts the effects of temporal urgency associated with the imminence of the disaster and the effects of limited shelter capacity for evacuees. In this section we summarize our key findings, discuss several methodological considerations, and describe implications for future work.

### Predictive, scalable Model of Collective and Individual Human Decision Making

Based on empirical measurements of the cumulative rate of evacuations as a function of the instantaneous disaster likelihood, we developed a quantitative model for decision making that captures remarkably well the main features of observed collective behavior across the 47 disaster scenarios. Moreover, we are able to capture the sensitivity of individual and population level decision behaviors to external pressure on resources (limited shelter capacity) and time (imminence of disaster). Systematic deviations from the model provide meaningful estimates of variability in the collective response. Our analysis uncovers a temporal evolution in individual behavior over the course of the experiment, indicative of increasing attention and swiftness of response, and consistent with the expectation that individuals learn from previous incidents.

Our model is not assumed to have a strict threshold form as in previous numerical studies [37], but uses rates to account for stochastic variability in behavior. Nonetheless, when fit with data from our experiment, the model exhibits qualitative threshold-like behavior that depends on multiple experimental variables.

Data from the experiment reveal significant heterogeneity in individual decision making patterns captured by significant variation in model parameter fits to participants. The results distinguish between high scoring individuals whose decisions to evacuate are strongly linked to a tight range of disaster likelihoods, versus others who exhibit significantly more variable decision making patterns and did not score as well in the experiment. Both the individuals’ overall success rate in the experiment and the decision making variables that model their behavior are correlated with heterogeneities in individual risk attitudes, as measured by established psychological tests.

These results suggest new directions for numerical modeling. For example, simulation studies that extrapolate decision making strategies identified in small groups to larger collectives could more accurately predict behavior in large scale populations and coalitions. Additionally, simple mathematical models are needed to better understand the tensions and tradeoffs identified in this experiment. Effects of competing broadcast and social information in collective decision dynamics have been investigated previously in a numerical simulation, where individuals were represented by nodes in a network, and obtained information from a broadcast source as well as neighboring sites in the network [37]. In that case, decision making was modeled as a threshold on an individual state variable representing opinion, and the opinion of each individual was updated based on a stochastic contact rule with the broadcast source (essentially a warning that the disaster was coming) and other individuals (who might or might not have received any information about the disaster). The results presented in this paper suggest important extensions to that model that (1) incorporate different types of information from broadcast and social sources, including an underlying physical process involving likelihood and urgency and (2) directly implement the individual decision model developed in this study rather than assuming the more simplistic update rule employed previously. Our current research is focused on the design of experiments that will better characterize the role of social information and network structure.

### Methodological Considerations

While no laboratory experiment can fully capture the tensions associated with a true disaster, known factors influencing human risk perception and urgency were accounted for wherever possible in the experimental design. These include both linguistic and visual elements, which are well studied in the psychology and risk literature. Examples include the use and representation of disaster likelihood rather than probability, as well as scores for each scenario represented in terms of a potential loss rather than a payoff for a scenario. Previous studies have shown that humans respond differently to losses than gains [62], [63], and are significantly more accurate in decision making based on data presented as likelihoods than on data presented as probabilities [64], [65].

The changing likelihood presented to the participants in this study represents the uncertain, and highly variable physical processes that govern the real time approach of natural disasters, such as wildfires or hurricanes [49], [58], [60], [85]–[87], and that ultimately result in either a “Hit” or a “Miss” for individual homeowners or communities. The existence of an underlying, quantifiable process for the disaster introduces objective parameters that govern volatility, difficulty, and uncertainty that can be varied in the experiment. Higher volatility, as well as variable time steps, leads to an outcome that is more difficult to predict. Based on the rules of the process, it is possible to calculate the likelihood of the disaster at each time increment (which is the only aspect of the process presented to the participants in this experiment, and it is presented at limited resolution), as well as the optimal evacuation decision (in the absence of shelter capacity limitations) [67].

The details of this process were deliberately hidden from the participants, who were only presented with the current estimated likelihood of the disaster hitting their community, updated at one second intervals. Our decision to obscure most of the details from the participants was based on observations of realistic disaster event scenarios where the public has access to limited information about the disaster likelihood. The complexities of geophysical events are commonly reduced to highly simplified trajectories and “likelihoods” when presented to the public whether it be the chances of rain, or the chances of a disaster [86].

In any behavioral experiment, it is of interest to compare participants’ actual behavior to optimal behavior from a profit-maximization perspective. In our experiment, the optimal evacuation time depends both on the volatility of the disaster process and on the potentially confounding actions of other participants. While the choice of an underlying stochastic process in principle allows for the calculation of a limiting theoretical optimal decision strategy [67], our results demonstrate that human behavior departs from optimality at a more primitive level. As previously discussed, even in the simplest cases where an optimal strategy is easily obtained (i.e., where there is no competition for shelter space, and the time of the possible disaster strike is known in advance), the participants still act sub-optimally. This result highlights the critical importance of uncovering predictive models of the suboptimal decision strategies that humans employ in real and laboratory settings.

### A Framework for Quantitative Analysis and Prediction of Human Behavior in Disasters

In the development and assessment of policy for disaster mitigation and response, human behavioral factors are often the least well quantified, understood, and modeled. Plans for evacuation based on broadcast communication and transportation alone can be rendered ineffective if humans do not act as expected. In retrospective analysis of data from recent events [49], [50], [58]–[60], prediction and planning for human social factors have been identified as the critical missing link in developing effective strategies to insure safety of the population as a whole. As a result, critical resources are diverted to individual crisis hot spots that might have been avoided with a more effective plan, and in many cases lives are ultimately lost.

These shortcomings motivate our investigations, which represent the initial steps in development of a comprehensive, predictive framework that incorporates human factors in policy and planning for disaster mitigation and response. Success in this area mandates an iterative approach that combines numerical modeling with controlled experiments and retrospective analysis of data collected from actual disasters. Our study uncovered multiple drivers of individual decision making behavior from competing information sources. The social network as a whole provided a source of information on shelter occupancy, inducing a sense of urgency in the population, while the topology of the network surrounding a given individual (i.e., the number of that individual’s neighbors) swayed the time spent engaging the social network. Despite these influences, individual participants spent the majority of their time consuming the broadcast information, and the disaster likelihood was the primary factor influencing decision making strategies in the population as a whole.

The observed tensions between the two sources of information are consistent with empirical observations of human behavior in real disasters. Outside of the laboratory setting, the likelihood of a disaster event is clearly a dominant factor in any decision to evacuate, and individuals spend a great deal of time gathering information from television and other media broadcast sources, even if updates are slow. However, social media and peer-to-peer communication networks are playing an increasingly important role in transmission of early warnings by on-site observers who may communicate observations informally via Twitter and Facebook [88] (e.g., news of a 2011 earthquake in the Washington D.C. area propagated faster on social networks than the seismic waves themselves [89], [90]). Furthermore, in some cases, such as developing countries, widespread access to broadcast networks may not be readily available, necessitating that policy makers rely on social means to communicate information updates. Future experiments will change how participants access information in order to investigate these situations, and elucidate the corresponding effects on behavior.

Additionally, in many (if not most) cases social factors underlie the decisions of individuals who evacuate early or fail to evacuate even when the disaster is upon them [48], [50], [60]. For example, families with small children tend to leave early, while caring for the elderly or reluctance to leave pets behind are often cited as reasons for not evacuating. These factors could be incorporated in future experiments using an explicit payoff structure that rewards collective decisions of neighbors in the social network. Another observed source of variation in evacuations during disasters can be traced to heterogeneities in age, health, isolation, and socioeconomic status within the population. These factors influence speed and access to transportation, as well as potential losses associated with assets at risk. Such sources of variation may be incorporated in our framework by introducing explicit heterogeneity in the loss matrix and in the scenarios accessible to a participant during the InTransit phase.

Finally, our work highlights the role that individuality plays in the decisions of participants and their effect on collective behavior. The distribution of risk tendencies in this experiment might be related to the demographics of the cohort studied here (UCSB undergraduates), and future studies utilizing different participant groups could be used to probe such a relationship. For example, it is reasonable to expect that older and wealthier individuals (e.g., homeowners) might be more risk averse in this domain than undergraduate students. Furthermore, participants who are explicitly trained in risk management and/or operate within different organizational structures (e.g., military officers) might employ different decision making strategies, and a group of such participants might by extension display a quantitatively different collective behavior profile.

Our combined use of a novel experimental paradigm and powerful theoretical modeling techniques to identify and quantitatively characterize individual differences in human decision making strategies in social groups could form a critical bridge to key work in the fields of social neuroscience [91] and neuroeconomics [92], [93], which seek to describe neurophysiological correlates of social and economic considerations driving human decision making. Indeed, human neuroimaging studies highlight the role of specific brain regions in economic choices and variations in decision strategies [94], [95]. Individual differences in these circuits could underlie behavioral decision phenotypes in healthy and diseased clinical populations [96], [97]. Uncovering neurophysiological predictors of decision dynamics in social groups would have far-reaching implications for disaster preparation and response, marketing, and homeland security.

### Development of Strategies to Mitigate or Manage Collective Evacuation Behavior

The ultimate goal of our investigations is development and testing of robust strategies for training and control of evacuations that account for human behavior and network topologies. These objectives may be incorporated within our framework across both broadcast and social channels. Broadcast information may include specific timing for public release of information, including likelihood updates and incentives as well as warnings and mandates for evacuation. In the peer-to-peer communication network, strategies for robust control and potential fragilities of collective behavior may be investigated through insertion of trained “leaders,” who make optimal decisions at different locations in the network, as well as through tracing the propagation of deliberately injected misinformation and poor decisions. Results obtained for these “designed” strategies may be compared to emergent leadership that might arise when the ranking and decisions of other individuals in the network is communicated through the social network, an inherent source of feedback which has been traced to the initiation of cascades in social decision making in a wide range of applications [21].
